# Supplementary material for: A network model of the barrel cortex combined with a differentiator detector reproduces features of the behavioral response to single-neuron stimulation
Source: PLoS Comput Biol. 2021 Feb 8;17(2):e1007831. doi: 10.1371/journal.pcbi.1007831 (PMC7895413; doi:10.1371/journal.pcbi.1007831)
Supplement: Attachment — Submitted filename: PCOMPBIOL-D-20-00502-reply-to-reviewers.pdf [file pcbi.1007831.s001.pdf]

Submission to PLoS Computational Biology:

"A network model of the barrel cortex combined with a differentiator detector reproduces features of the behavioral response to single-neuron stimulation"

(PCOMPBIOL-D-20-00502)

Davide Bernardi, Guy Doron, Michael Brecht, Benjamin Lindner

Reply to Editors and Reviewers

Dear Dr. Bernardi,

Thank you very much for submitting your manuscript "A network model of the barrel cortex combined with a differentiator detector reproduces features of the behavioral response to single-neuron stimulation." for consideration at PLOS Computational Biology.

As with all papers reviewed by the journal, your manuscript was reviewed by members of the editorial board and by several independent reviewers. In light of the reviews (below this email), we would like to invite the resubmission of a significantly-revised version that takes into account the reviewers' comments.
Note that a common theme across reviews consists of questions the readout aspect of the model. This aspect clearly needs some serious focus in the revision process. In that process, please also be sure to clarify how this work represents a significant advance relative to the previous papers of this group.

We would like to thank the Editors and Reviewers for the time and effort devoted to our manuscript, and for providing us with constructive and useful feedback.

We revised our manuscript extensively, and modified some aspects of the model according to the reviewers' comments. The most significant changes to the resubmitted version concern the readout, which was improved owing to the reviewers' suggestions, better explained and motivated.

In the introduction and discussion of the revised manuscript, we also better emphasize how the present work differs from previous studies that were published in Phys. Rev. Lett. (2017) and Phys. Rev. E (2019). These studies were inspired by the single-neuron stimulation experiments of Houweling and Brecht published in *Nature* (2008), and, for this reason, they can be related to the present work. However, these theoretical papers only dealt with the problem of how the stimulation of a single neuron can be detected in a large spiking network that retained only some general properties of the cortex. The focus of the present study is to find a possible explanation for how the response to the single-neuron stimulation depends on the properties of the injected current, a question studied by later experiments (Doron et al. *Neuron* 2014), and that was completely neglected in our previous work. We remark that these findings place many constraints that are rather difficult to satisfy simultaneously, and

that are not fulfilled by our previous attempts. Indeed, the model we consider here is strongly different both in the properties of the network, which is much more complex, and in the readout strategy. In this regard, we write in the revised introduction (l. 40-63):

> Especially challenging for theoreticians are the results of the nanostimulation experiments in the barrel cortex of behaving rats [6] which demonstrated striking dependencies of the behavioral response on the properties of the stimulating current. The response does not depend on the duration of the stimulus, it depends weakly on its intensity, and strongly on its irregularity: the response is greatly enhanced if the current varies irregularly within the stimulation window instead of being held constant.
>
> None of these findings can be explained by the generic setups previously investigated in the context of single-cell stimulation [25, 26]. The results in ref. [6] represent a challenging and complex set of constraints that are difficult to fulfill at the same time, especially for a model based on spiking network models, which are notoriously expensive to simulate and hard to treat analytically.
>
> Here, we tackle this problem by studying a computational model that includes more biological details of the barrel cortex: a network with three distinct cell types (one excitatory neuron type and two distinct inhibitory interneuron classes), which are all modeled as integrate-and-fire neurons endowed with adaptation currents, short-term synaptic plasticity for chemical synapses, which can be facilitating or depressing according to the cell types, and electrical synapses (gap junctions). A further crucial difference to our previous studies is that we compare two ways of reading out the network's response to the stimulation of one randomly selected neuron: one based on the integration and one based on the differentiation of the network's activity. We also show that a basic excitatory-inhibitory circuit can be used to approximate the differentiator, and demonstrate that the response of this so-called *differentiator network readout* is in several key aspects similar to the behavioral response observed in the experiments in ref. [6].

In the following, we provide a detailed answer to all comments made by the three reviewers and refer to the appropriate changes in the manuscript. All figures except figs. 1, 5, and 6 have been edited or replaced, and two new figures have been added. We provide a version of the manuscript in which additions and changes are highlighted in red.

Reviewer #1:

This paper examines the contribution of single-unit stimulation in a detailed computational model of barrel cortex.

MAJOR POINT (1)
A fundamental advantage of working with computational models is not just the ability to capture experimental results, but, going further, to test the robustness of these results to alterations in the design of the model itself. After all, in some respect this is something you can really only do with computational models, so you have to take advantage of it wherever possible. Unfortunately, authors present only limited advances in this respect.

We would like to thank the reviewer for the time devoted to our manuscript and for the constructive comments, which helped us improve the model and better test the robustness of our findings. We did our best to follow the reviewer's suggestions, as detailed below.

> ***R. #1:*** *Here are three areas we would like to see extended:*
> *(1) line 116: how large of a subset of RS cells are fed into the readout? how much does it affect readout performance (i.e., effect size)?*

In the original submission, the size of the readout set was 1000, which corresponds to one half of the RS population. We apologize for giving this information only in an implicit way. In the revised model, the readouts can receive input from all three neuronal types, as suggested by the first and third reviewer (see the comments below). Accordingly, we investigated systematically the dependence of the readout performance as a function of the size of *all* three readout populations. The results are summarized in the new fig. 10 and discussed in a new subsection ("Roles of cell types and delay difference on readout performance" l. 527-571), in which the roles of the cell types are discussed. Importantly, the main effects are robust, as the DNR is consistently more efficient in detecting the irregular stimulus, whereas results from the IR show the opposite behavior. In the revised manuscript, we discuss the new fig. 10 as follows (l. 527):

> After having shown that the DNR is most consistent with the experimental results, we will now explore how the readout performance depends on the size of the three readout populations, i.e. the effect of each neuron class on the detectability of the single-neuron stimulation. The simulation results are summarized in fig. 10. For brevity, we will focus on the IR (first row in fig. 10) and DNR (second row in fig. 10), and use only the 400 ms stimulus (represented by blue circles) and irregular stimulation (red squares).
>
> First, we can systematically vary the size of the RS readout population. Intuitively, the effect size should increase, the more RS neurons are fed into the readout. Simulation results demonstrate that this is indeed the case both for the IR readout (fig. 10A) and the DNR readout (fig. 10B): the effect size grows monotonically with the size of the RS readout population.
>
> Next, we can study the effect of a systematic variation of the FS readout population's size. In fig. 10C (D), it can be seen how the effect size measured by the IR (DNR) decreases when the FS readout population is enlarged. The strong self-inhibition of FS cells tends to stabilize their firing rate. Hence, despite the strong SOM response within the BCN, the average firing rate of FS cells within the BCN is, on average, only slightly reduced during the single-cell stimulation. However, due to the strong output weights of FS cells, they have a considerable effect on the readout activity. Because they disinhibit the readout during the stimulation, the effect of FS cells on the readout is antagonist to that of RS cells.
>
> The readout performance is improved by feeding more SOM neurons into the IR (fig. 10E). The readout performance of the DNR is generally enhanced by enlarging the number of SOM readout cells (fig. 10F). However, the effect size tends to saturate (for irregular stimulation) or even slightly decrease when the size the readout population approaches the entire SOM population (200 cells). One possible reason may be stronger cross-correlations between SOM cells.
>
> As a final comment to fig. 10, we note that the DNR detects the irregular signal with a higher accuracy over the entire range of parameters, while the IR readout is almost always better at detecting the regular signal. In this respect, these

findings are robust, and the DNR is a stronger candidate than the IR as a possible readout mechanism which is in line with the experimental results.

R. #1: *(2) how does performance deteriorate with delta_T<10 ms?*

We investigated the dependence of the readout performance as a function of the delay difference ΔT in the new Fig.11. The results in Fig. 11 are commented as follows (l. 558):

> One further crucial parameter for the operation of the DNR is the delay difference of the two input pathways (ΔT). It is therefore important to check how robust our results are with respect to this parameter. As shown in fig. 11, the effect size measured upon irregular stimulation is larger than that observed for regular stimulation also for when ΔT is decreased below 10 ms, although it decreases in magnitude in both stimulus conditions. When ΔT is further decreased below 5 ms, the effect size reaches zero and becomes negative for the regular stimulus. Hence, the qualitative picture is robust for $\Delta T \gtrsim 5$ ms, as the weaker effect could be enhanced by leveraging, for instance, the dependencies seen in fig. 10.
>
> We note here that it is difficult to try out all possible parameter combinations due to the high computational cost of simulating the network for multiple conditions and a large number of trials, which is needed to measure a possibly small effect size reliably. The simulation results shown in figs. 10 and 11 required about two weeks on a computing cluster with more than 200 cores.

R. #1: *(3) line 314: would be nice to show a full curve of stim duration vs. number of spikes evoked w/ SEM. I expect this result should depend on the sparsity of connectivity, but should be largely independent of system size.*

The new fig. 7B at p.16 shows the number of evoked spikes in the target neuron vs. stimulus duration in the model and the experimental results. In the revised manuscript, we state more explicitly (l. 354-355) that we plot the spikes evoked in the stimulated neuron, and not in the entire network, which are experimentally unknown. Because the single-cell stimulation represents a strong suprathreshold input, the neuron fires tonically during the stimulation and the properties of the recurrent network input and background noise play only a minor role. For instance, we show here the spike-count vs. duration graph

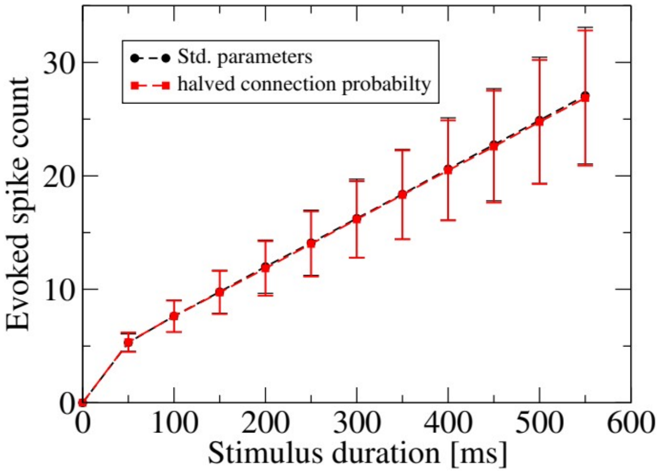

for the standard parameters (black circles) and for a network of doubled size and halved connection probabilities (double connection sparsity). The two plots are almost identical.

***R. #1:** MAJOR POINT (2)*
The model assumes that SOM and FS populations from the BCN network play no role in the readout of sensory information. While the effect of a single FS spike on FS population is small, SOM cells are strongly excited in response to stimulation. Therefore, could they contribute to readout accuracy? This could be easily addressed by feeding SOM to your DR or DNR readouts, and comparing the accuracy with readout from RS only.

Thank you for this suggestion. In the revised model, the readout receives input from all inhibitory populations and their effect has been investigated systematically in Fig. 10. Indeed, the SOM cells can enhance readout performance significantly, whereas FS have an opposing effect (see also Fig. 10 and main text on p. 21-23 of the revised manuscript, which has been quoted above).

***R. #1:** MAJOR POINT (3)*
When comparing the models (IR vs. DR vs. DNR) to experimental data, authors are strictly focusing on a measure of effect size. However, just as interesting, is the standard deviation (sigma_hat_x(t)) of the model in response to RS stimulation. This standard deviation is much larger for DNR than DR in all simulations from Figs.7,8,9. Which model fits the experimental data better? If this cannot be validated with available data, please discuss how it could be addressed.

Unfortunately, we cannot directly compare the mean and standard deviation of the readout population to experimental data, because this comparison would require recording the full spiking activity of the local network performing the readout. In principle, an experiment in which the single-neuron stimulation is performed while recording the extracellular activity of the readout network could provide such information. One major problem is, however, that we currently know neither where the readout circuit is nor whether it is a single localized network, or rather a distributed computation. We write now in the revised discussion:

> [l.629] ...the readout part of our model represents a generic downstream area and may or may not be located within a specific layer, or within the barrel cortex itself. One hypothesis could be that the stimulated cell resides in deeper layers and that the readout network is located within the supragranular layers. At the present state, however, there is no specific element in support of this conjecture before others, including the possibility that the readout computation is not localized in a single population, but rather distributed across several downstream areas.
> [...]
> [l.674] One way of identifying candidate areas as a readout network would be an experiment in which the juxtacellular stimulation is performed in parallel with multi-electrode array extracellular recordings of the pooled activity of the local network. In this way, one could directly access $\mu_{\hat{X}}(t)$ and $\sigma_{\hat{X}}(t)$ (the deviation from the spontaneous state of the pooled local activity's mean and standard deviation, and thus extend the experimental data that can be compared to our model results beyond the change in the probability of a behavioral response, i.e. the effect size.

R. #1: *Further, what other comparisons could be make between DR and DNR with respect to to barrel cortex recordings?*

Since the DR is based on a mathematical operation (a subtraction) performed on the subthreshold activity of one cell, it is hard to imagine that it could be implemented *exactly* by a neuronal circuit. The DNR was introduced as a simple way in which a downstream population could approximate this operation. Hence, the difference between the two readout schemes DR and DNR is more of a conceptual nature and this is why we cannot think of a way of distinguishing the two readouts by means of an experiment.

R. #1: *MINOR POINTS*
The legend of fig.2 is redundant, first line: A cell selected at random from the barrel cortex network (BCN) is selected at random. (Yes, we get it, it's selected at random...)

This sentence was edited and is no longer redundant, thank you for pointing this out.

R. #1: *The way in which the detection problem is framed in Fig.3 is highly reminiscent of the Tempotron problem (Gutig & Sompolinsky 2006 Nat Neurosci). However, this work is not cited. Please cite this paper and briefly address any similarity with your work (in terms of the problem, and not the learning algorithm of Gutig, of course, since you don't have any long-term plasticity).*

The Tempotron paper is now cited in the discussion, in which we elaborate in more detail how learning rules similar to the Tempotron can, in principle, make use of the much larger information contained in the full spiking pattern of the network (or of the readout subset), but are difficult to apply to our problem, as the training is done via microstimulation and is not cell-specific. In this regard, we write in the discussion (l. 703):

> Except for some minor technical differences, interpreting the IR activity as the voltage of a single “grandmother” neuron renders the detection problem somewhat reminiscent of the “Tempotron” readout [70], which can learn to respond to specific spiking patterns. Theoretical studies have shown that one single spike can be enough to perturb the firing pattern of a spiking network [71, 72], and it has been argued that the same may hold for the actual cortex as well [9]. Since the nanostimulation elicits multiple spikes in the targeted neuron, it is likely that some learning algorithm sensitive to precise spiking patterns, akin to the Tempotron, could easily detect the single-cell stimulation and achieve a better performance than our readout, which simply considers the pooled activity from a fraction of the local network activity. In fact, it has been shown that a simple classifier can discriminate highly correlated inputs to a spiking network which exhibits chaotic spontaneous firing activity [73]. Although some explicit training of the readout weights could indeed drastically increase the effect size and the experimental subjects did undergo a training phase before the single-neuron stimulation sessions [4, 6], we chose not to explicitly train the readout to detect specific cells, because the training in experiments was performed by employing microstimulation pulses, which intricately affect a large area rather than a specific cell directly [74–77]. Our readouts, that rely on the pooled activity of the local network, discard a lot of

information that can be stored in precise spiking patterns, but they are also more robust and, most importantly, insensitive to the choice of the specific neuron.

***R. #1:** While Fig.3 begins by describing the detection problem as a boundary-crossing problem with a lower boundary set on the readout activity, we learn later that in fact both a lower and an upper boundary can be used (unless I am misunderstanding...) (i.e., line 178). There is a bit of confusion about whether any (or all) of your different readouts (IR, DR, DNR) can indeed make use of both an upper and lower boundary. Please clarify.*

In our model, the IR uses a lower boundary, whereas the DR and the DNR use only an upper boundary. This choice was made to obtain a positive effect size for all detectors, which is consistent with the average effect size observed in the experiments. In the revised manuscript, this point is better clarified both in the main text (l. 173-178) and in the revised fig. 2 (in which we indicate explicitly the choice of lower or upper boundary for each readout) and in the new fig. 3 (which depicts both cases of lower and upper detection crossing):

> Trajectories computed from eq. (4) are used in combination with a simple threshold detector, as in the previous case. Note that the DR, however, uses an *upper* detection threshold θ_+ , as shown in fig. 3B. Black and red trials plotted in fig. 3B represent again a catch and stimulus trial, respectively. They both exceed the chosen threshold at least once within the detection window. Therefore, both trials contribute to false positive and correct detection rates, respectively. These rates are defined as in the previous case, except that here an upper threshold is used:
> [...]
>
> [l.174] There is nothing profound in the choice to endow the IR with a lower detection boundary and the DR with an upper detection boundary. The essential reason is that, in this way, we obtain a positive *effect size* (defined below), as it was observed in the experiments. In other words, this combination works for the readout schemes we consider here.

***R. #1:** Figure 1, last line of caption: no need to repeat the values of mean firing rates since they are already listed in the main text (line 188).*

This redundancy was removed from the caption.

***R. #1:** Figure 5: legend should describe what are the shaded areas in RS, FS, and SOM populations. this is not entirely clear from text – does it refer to connectivity, or some measure of network activity? All I see is " spikes of the stimulated RS neuron reach a large fraction of the FS population" but I don't understand whether this refers strictly to a connection probability or to spiking activity, e.g., in RS causing depolarization in a number of FS spikes. If the latter is true, then not sure how you establish such causality.*

The shaded areas refer only to the connectivity, as now explicitly mentioned in the caption. Furthermore, we edited the text (l. 247, 251, 254) to emphasize that the discussion is limited to the effect of direct synaptic connections. An additional sentence (l. 274-276) in the main text underscores

the fact that the entire purpose of fig.5 and of this subsection is to give a coarse picture of the firing-rate response of the network, which nevertheless is in qualitative agreement with results from the experimental literature:

> Clearly, this description of how the single-neuron stimulation affects the firing rate of the network is greatly simplified, and it gives only a coarse picture of the actual network's response. However, these observations are consistent with in vitro experiments showing that...

R. #1: *line 417: typo: These stimuli, in accordance with the experimental procedure [6, 47], is a. *are a**

This has been corrected. Thank you.

R. #1: *Figure 7 panel: First row is what? you state "standardized deviation from the spontaneous value of the time-dependent mean readout activity" but I thought this was just the mean, i.e., Eq.13. why "standardized deviation"? Same question for the second row, why is it "standardized deviation"?*

We meant that the deviation from the spontaneous value of mean and standard deviation of the readout activity are shown in units of the spontaneous standard deviation of the readout activity, i.e. eq. (13) for the first row, and eq. (16) for the second row. In the captions of fig. 7-9, we now use a more precise formulation. We also have specified these statistics in more detail when introducing them on l. 301-303:

> More precisely, we will consider first the time-dependent deviation from the spontaneous value of the readout activity $A_X(t)$ (here X= ir, dr, dnr, as defined above), normalized by the spontaneous standard deviation:

and on l. 306-307:

> The time-dependent standard deviation of the readout activity is defined in a similar way (again a relative measure, given in multiples of the spontaneous standard deviation):

R. #1: *The caption of Fig.10 needs more explanation; are these different cells or the same cell over several recordings?*

These are recordings from different cells. In the revised manuscript, we show in the new Fig.12A four of the six curves of fig.10 of the first submission. The caption of the new figure states that the curves refer to different cells.

Reviewer #2:

The authors present a large scale LIF model of cortex that contains pyramidal cells as well as SOM and PV inhibitory cells. They tune the network to lie in a balanced state and then stimulate single pyramidal neurons in order to mimic recent experiments that are in the barrel cortex of mice. Since the experiments indicate that single neuron stimuli can affect the behavior, the authors created this large scale simulation and then try to design a way to detect the presence of the stimulus. Thus, they build a second network that takes one of three forms: integrator, differentiator (via delays) and differentiator network. There are merits in the paper, but also some flaws that need to be addressed. The present work is not terribly different from the authors previous efforts in 2017, 2018 other than applying them to the barrel. The fit of the data is quite good for the DR and DNR but we don't really know if the IR was optimally fit.

We thank the reviewer for the time and efforts devoted to our paper. We appreciate that the reviewer found some merits in our work. We hope that we made a convincing point in the revised introduction, which we quoted on p. 2 of this reply, that both the goal and the methods of our present manuscript are substantially different from our previous work.

We are especially thankful to this reviewer for the important hints to the literature, the incorporation of which has enriched the paper. Finally, we are grateful to the reviewer for the suggestion to test the effect of nonstationarities directly; the corresponding results have provided additional support for the differentiator-readout scheme.

Regarding the question of whether the model was optimally fit, we would like to add the following. Finding a good agreement between all data and simulations with the DNR required, of course, some parameter adjustment. However, no optimal fitting has been done for *either* detector prescription, simply because it is not feasible. In fact, a reliable measurement of the effect size requires simulating thousands of trials for each experimental condition, and a systematic search in a parameter space with over 50 dimensions is, hence, impossible, even if the space is partially constrained by the available experimental literature on the cellular and network properties of the barrel cortex, and by the properties of the network's spontaneous activity. Only a few attempts were needed to have the DNR reproduce the experimental results. On the contrary, we repeatedly tried without success to explain the experimental data by using an integrator readout scheme only. Furthermore, the results discussed in our reply to Reviewer #1 and shown in the new figs. 10, 11, and 12 support even more the superiority of the DNR in describing the experimental results.

R. #2: *Major Points:*

A number of papers related to barrel circuitry inhibition and differentiation are missing from the discussion here. The authors should discuss papers by Pinto, Simons and their coauthors as well as the old integrate and fire models of barrel cortex by Kyriazi and Simons. All these papers are concerned with the understanding of how inhibition is able to make the barrel responses into more like a differentiator than an integrator and thee authors wrote many papers on this several decades ago. Since the key feature in the present paper is a so-called differentiator circuit, and these old papers tuned their model to fit experimental data from layer 4 rat barrels, it seems that they need to be acknowledged.

We apologize for the oversight of these relevant references, and we thank the reviewer for pointing this out. These pioneering studies elegantly uncover mechanisms through which the barrel system in its entirety can perform the differentiation of the sensory input, and are indeed important references in this context that are now properly acknowledged. We still would like to note the different objective of our study, which is not concerned with the bulk response of the barrel cortex to a "regular" stimulus, that is, a stimulus reaching hundreds of cortical neurons via the sensory afferents, but with the response to a stimulus with *cellular resolution*. How such kind of stimulus can be detected and processed by the barrel cortex (and successive stages in the information flow) is still an open problem, and is the main question addressed by our manuscript. In the revised discussion, we now write (l. 643):

> Among the biological processes included in the model, short-term depression and spike frequency adaptation could be expected to oppose slow changes in the input. However, our results indicate that these mechanisms may not suffice to explain the data if an integrator readout (IR) is employed. If a differentiator readout (DR or DNR) is used instead, simulation results are in agreement with the data. In this respect, our results fit into the picture emerging from the classic experimental and modeling studies showing that the barrel system as a whole responds to whisker movements more as a differentiator rather than as an integrator [56–65]. These studies showed with an elegant combinations of experiments and computational modeling how the on- and off-responses to whisker deflections, which are already present in the thalamic response, are sharpened within the barrel cortex, and elucidated the key role of inhibition, and, in particular, of cross-whisker inhibition. Refs. [56–65] already established the key role of inhibitory neurons in making the barrel system respond more like a differentiator than an integrator, and they did not postulate the existence of a further readout circuit, which performs the differentiation. Our model suggest that at least one further processing stage is needed to explain the experiments from ref. [6], which, in these earlier work, was not needed to explain the response properties of the barrel cortex. It must be noted, however, that we consider here a completely different situation, i.e. that of an extremely weak (at the network level) input that is delivered directly into the cortex. This stimulus, therefore, overrides the preprocessing performed by the afferent sensory pathways, which contribute significantly to the differentiation operation performed by the whisker sensory system. It was, hence, a still open question whether a similar kind of sensory processing that applies to a volley of correlated inputs such as those received from thalamocortical projections upon whisker deflection [56, 64, 66, 67] would apply to the experimental situation of refs. [4, 6]. Our model suggests that this might be the case, provided that a second downstream area acts as a differentiator.

> ***R. #2:*** *Is their network a single barrel? Or is this supposed to be the full cortex. I suspect the former but in any case they should use more precise language. Is this layer 4 or supposed to be some other layer?*

The spiking network model we consider (the BCN) is even smaller than a single barrel, and represents the surroundings of the stimulated cell. Hence, the BCN lacks a layered structure, because of its small size, and a layer-specificity, because in the experiments there was no reliable control of the depth of the stimulated cell, and no specific layer was targeted. Hence, we deliberately chose not to specify a single layer. The three cellular types of our model are present and, despite fluctuations in the relative

proportions, are well represented in all layers (except for layer 1, see Meyer et al. PNAS 2011). In the revised manuscript, we state these facts more precisely both when the model is first introduced (l.70):

> The recurrent network model represents the surroundings of the stimulated cell in a radius of about 200 µm, in which connection probabilities can be considered as constant [27, 28]. Taking into account the estimated neuron density in the barrel cortex [7], a sphere with a radius of 200 µm contains about N = 2600 neurons, which we therefore take as the network size. Although this network size corresponds to a fraction of one barrel, and an even smaller fraction of the entire barrel cortex, this part of the model will be referred to as "barrel cortex network" (BCN); we stress that the BCN only describes a generic subnetwork within the barrel cortex and is not tailored to one specific layer, in line with the single-cell stimulation experiments [4, 6], which did not target one layer in particular. The BCN consists of three neuronal populations: excitatory regular spiking cells (RS), inhibitory fast-spiking (FS) cells, and somatostatin-expressing low-threshold spiking (SOM-LTS) inhibitory neurons. These three cell types account for a large fraction of the neurons found in the barrel cortex (for instance, they account for about 99% of layer IV [29], but numbers in other layers are similar [30]). All neurons are modeled as one-compartment leaky integrate-and-fire (LIF) neurons. Besides leak conductance and spike generation, several other biological mechanisms are modeled, according to the cell type.

as well as in the revised Discussion (l. 622):

> The spiking network model (BCN) we constructed in this study incorporates several features of the barrel cortex, and its parameters were consistent with the experimental literature. The BCN was chosen to represent only the immediate surroundings of the stimulated cell and its size is about one sixth of one standard cortical column [7]. Being too small, our network does not have a layered structure. Furthermore, it does not represent one specific layer within one "cortical column" because there was no clear layer specificity from the experimental side, and, presumably, cells from different layers were stimulated. Also the readout part of our model represents a generic downstream area and may or may not be located within a specific layer, or within the barrel cortex itself. One hypothesis could be that the stimulated cell resides in deeper layers and that the readout network is located within the supragranular layers. At the present state, however, there is no specific element in support of this conjecture before others, including the possibility that the readout computation is not localized in a single population, but rather distributed across several downstream areas.

***R #2:** The abbreviation BCN is never defined.*

In the original submission, the abbreviation was introduced only after its first use, we apologize for this mistake. This has been fixed in the revised manuscript, in which BCN is defined at the proper place (l. 77).

R #2: *It is unclear to me what makes this barrel cortex as opposed to any other cortex.*

The three cell classes and the main biological mechanisms of the neurons and synapses of our network model (adaptation, synaptic facilitation/depression, gap junctions...) are indeed present in most cortical areas. However, we did refer to the available experimental literature on the rat barrel cortex to choose the range in which all cellular parameters are heterogeneously distributed, and to assign connection probabilities and synaptic dynamics. Some of these characteristics are similar in other cortical areas, so that the general traits of the BCN could apply to other cortical systems. Although the BCN is strongly inspired by the available knowledge on the barrel cortex, it might be a fair model for other cortical regions. If this is true, our model might suggest that the single-cell stimulation could be detected (after specific training) also in other cortical areas, which is still an open problem. To reflect these ideas, we now write in the discussion (l. 636):

> It is still unclear whether specific anatomical properties of the barrel cortex make it particularly sensitive to the single-neuron stimulation. In this regard, although we did use the available experimental literature on the rat barrel cortex as reference to choose the range in which all cellular and synaptic parameters are heterogeneously distributed, some of these characteristics are similar in other cortical areas. Hence, the general traits of the BCN could describe also other cortical systems. If this is the case, other cortical areas might be sensitive to the single-cell stimulation.

R #2: *The whole concept of a readout circuit seems really contrived and weakens the proposed model substantially. In the old work of the Simons group, there was no need for such a circuit, it was all contained in the basic barrel network. I am having a lot of trouble figuring out what part of the barrel ctx each of these modules represents as they are not specified eg as layer IV or what. The primacy of this aspect of the model makes it foubtful to me that it will be a good model for cortex as it seems to be an artifice that is needed to make sense of the extremenely noisy outputs of the model.*

As already mentioned above, the earlier works of the Simons group elegantly demonstrated how whisker stimulation is processed by the whisker system culminating in the barrel cortex. However, they also showed how the input to the barrel cortex already has a clear on- off-response, as opposed to a sustained response. In other words, upstream populations already contribute to the differentiation. Here, we consider a radically different situation, *i.e.* that of a short stimulus delivered *in vivo* directly to one neuron in the barrel cortex. It is, therefore, not surprising that further processing stages are required to obtain an agreement with the experimental observation. Furthermore, because the barrel cortex itself is a primary sensory area, it is rather implausible that it can either represent a decision or directly trigger a motor response. Hence, it is a likely assumption that (at least) a further neuronal population must be involved in the single-cell stimulation detection task. These issues are now addressed in the revised discussion as follows (l. 650):

> These studies showed with an elegant combinations of experiments and computational modeling how the on- and off-responses to whisker deflections, which are already present in the thalamic response, are sharpened within the barrel cortex, and elucidated the key role of inhibition, and, in particular, of cross-whisker inhibition. Refs. [56–65] already established the key role of inhibitory neurons in making the barrel system respond more like a differentiator than an integrator, and they did not postulate the existence of a further readout circuit, which performs the

differentiation. Our model suggest that at least one further processing stage is needed to explain the experiments from ref. [6], which, in these earlier work, was not needed to explain the response properties of the barrel cortex. It must be noted, however, that we consider here a completely different situation, i.e. that of an extremely weak (at the network level) input that is delivered directly into the cortex. This stimulus, therefore, overrides the preprocessing performed by the afferent sensory pathways, which contribute significantly to the differentiation operation performed by the whisker sensory system. It was, hence, a still open question whether a similar kind of sensory processing that applies to a volley of correlated inputs such as those received from thalamocortical projections upon whisker deflection [56, 64, 66, 67] would apply to the experimental situation of refs. [4, 6]. Our model suggests that this might be the case, provided that a second downstream area acts as a differentiator. The involvement of further processing stages is also reasonable if one takes into account that the barrel cortex is part of a primary sensory area, and that it is, therefore, implausible that it can encode a decision or elicit a motor response directly. It is, indeed, likely that at least one more processing stage is involved in the difficult task of detecting and reporting the single-neuron stimulation.

***R #2:** Minor points*
--- Page 9 ---
So is jread synaptic depression. I don't understand how it is implemented in the context of the readout neurons

The weights used for the synaptic filtering of the IR and DR activity are depressing, and they are treated as any other synapse in the network. This point is now better explained when the readouts are introduced (l. 148):

> Consistent with the idea that this readout is performed by other brain areas, and that synapses projecting to other brain areas are also likely to undergo short-term depression, the dynamic weights $J_{read,i}(t)$ are depressing and obey the same equation as all other weights in our model, i.e. eqs. (34) and (35). In other words, the readout activity $A_{ir}(t)$ can also be interpreted as the membrane potential of one "grandmother" neuron without fire-and-reset mechanism, which receives synaptic input from the readout sets only. This synaptic input is treated as any other synaptic input of the corresponding type in the network, with the only exception that readout weights from S^{SOM} to the readout are on average 20% stronger than those from the SOM to the RS population within the BCN.

R #2: *--- Page 12 ---*
Again, I see nothing here that points specifically to barrel cortex.

We believe that this point has been clarified above.

***R #2:** I recall some old papers by Latham that looked at the effects of deleting or adding a single spike to a balanced network which I think they include in the references. They should discuss the PRX 2012 paper by Monteforte and Wolf who describe similar effects.*

In the revised manuscript we cite the PRX paper by Monteforte and Wolf and discuss how our readout sacrifices the information contained in the spiking patterns of the network (drastically affected by the single-spike perturbation considered by these studies) for the sake of robustness and insensitivity to the stimulated cell's identity, which is crucial to model the experiments we consider. In this regard, we write in the revised discussion (l. 706):

> Theoretical studies have shown that one single spike can be enough to perturb the firing pattern of a spiking network [71, 72], and it has been argued that the same may hold for the actual cortex as well [9]. Since the nanostimulation elicits multiple spikes in the targeted neuron, it is likely that some learning algorithm sensitive to precise spiking patterns, akin to the Tempotron, could easily detect the single-cell stimulation and achieve a better performance than our readout, which simply considers the pooled activity from a fraction of the local network activity. In fact, it has been shown that a simple classifier can discriminate highly correlated inputs to a spiking network which exhibits chaotic spontaneous firing activity [73]. Although some explicit training of the readout weights could indeed drastically increase the effect size and the experimental subjects did undergo a training phase before the single-neuron stimulation sessions [4, 6], we chose not to explicitly train the readout to detect specific cells, because the training in experiments was performed by employing microstimulation pulses, which intricately affect a large area rather than a specific cell directly [74–77]. Our readouts, that rely on the pooled activity of the local network, discard a lot of information that can be stored in precise spiking patterns, but they are also more robust and, most importantly, insensitive to the choice of the specific neuron.

R #2: *the authors say:*
"Furthermore, their average amplitude is smaller if compared to other connections and they are strongly depressing, so that the direct effect on the overall firing rate of the RS population is small."
How can you tell that the effect is small without excluding the strong SOM inhibition?

This sentence has been changed to (l. 249):

> Furthermore, their average amplitude is smaller if compared to other connections and they are strongly depressing, so that - neglecting indirect multisynaptic paths - the *direct* excitatory effect on the overall firing rate of the RS population is small.

We also added a sentence to the end of this subsection, which emphasizes that this entire subsection was only a coarse, approximate description of the actual response of the network (l.274):

> Clearly, this description of the effects of the single-neuron stimulation on the firing rate of the network neglects is greatly simplified and gives only a coarse picture of the actual network's response. However, these observations are consistent with *in vitro* experiments showing that...

R #2: *--- Page 20 ---*
They talk about the readout network being trained. How would this be accomplished.

Modeling the learning process is a very elaborate problem, which is outside the scope of our manuscript. However, we can speculate that either an algorithm akin to the Tempotron rule (see above), or a Hebbian paradigm might work. We write now in the discussion (l. 723):

> In the construction of our model, we implicitly assumed that the training had already occurred and that it resulted in the formation of the differentiator circuit. It is possible that training the readout to detect the microstimulation with a suitable learning rule would produce a detector that is also more efficient in detecting the single-cell stimulation than the simple differentiator considered here. To this end, one could employ, for instance, a Tempotron-like rule, as mentioned above, or a Hebbian paradigm that pairs the microstimulation with the response of the readout population connected over several possible paths to the BCN. Beside the learning rule, however, an important problem would be how to construct a proper model for the complex, and only partially understood, cascade of events triggered by cortical microstimulation.

R #2: *Also, again, I really don't see where this readout network sits in the anatomy of the barrel cortex.*

We hope to have adequately discussed this issue above.

R #2: *They say:*
"A readout that integrates spiking in a sliding time window would experience difficulty in distinguishing the possibly small changes ... "

This seems easily testable in the model with very little work. I think the authors should show this is true rather than saving it for another paper.

A thorough investigation of this important question would require a proper way of modeling nonstationarities, and introduce radical changes in the entire model. In the revision, we do address this interesting problem in the new subsection "Robustness of readout mechanisms against slow input non-stationarity" (l. 572-603) under the simplifying assumption that nonstationaries are slow enough that they appear constant on the scale of one of our simulation trials (which last about 2 s in total). The results seem to support our original conjecture. We write (l. 573):

> The above results show that the DNR is more consistent with the experimental results. However, the effect size measured by the IR is larger in magnitude in most cases, except for the case of irregular stimulation, in which the DNR is only slightly better. This observation raises the question of why the animal should opt for a detection strategy which tends to detect the signal less often, considering that experimental subjects were rewarded upon successful detection. One possible answer, which we will briefly explore in the following, is that the DNR might be more robust with respect to slow fluctuation of the background input. It is possible to get a feeling for these slow non-stationarities by looking at fig. 12A, which shows the spontaneous firing rate of some cells over long justacellular recording sessions. The strong changes in firing rate are mostly unrelated to any stimulation event. A readout that integrates spiking in a sliding time window might experience

> more difficulties in distinguishing the possibly small changes induced by the single-cell stimulation from the strong background variations, whereas a differentiator readout might still separate the timescales of stimulus and background noise.
>
> Although a thorough investigation of this hypothesis goes beyond the scope of this study, in this last subsection we will explore this conjecture by adding a "static modulation" to the network background input. The basic idea is that a slow modulation of the network input will look nearly constant, if observed for the total duration of one of our trials (≈ 2 s). More precisely, in each trial we randomly draw $r_{\mathrm{ext,th}}$, one of the parameters that sets the global intensity of the external background noise (see Methods). Each sample is drawn from a lognormal distribution with constant mean (equal to 10 Hz, as in the simulations above) and standard deviation $\sigma_{\mathrm{r,ext}}$ (where $\sigma_{\mathrm{r,ext}} = 0$ corresponds to the situation considered so far, *i.e.* no background noise modulation). Figure 12B shows the effect size measured by the IR (light blue diamonds) and DNR (green triangles) upon increasing $\sigma_{\mathrm{r,ext}}$ for the case of 400 ms regular stimulus. Both detectors are affected by the additional noise in the background input. However, the effect size measured by the IR drops faster and becomes eventually smaller than the effect measured by the DNR. For the case that an irregular stimulus is applied, fig. 12C shows that both detectors suffer a performance loss when $\sigma_{\mathrm{r,ext}}$ is increased. However, the performance loss is stronger for the IR than for the DNR, which measures a larger effect size over the entire range of $\sigma_{\mathrm{r,ext}}$ considered here.

Furthermore, we write in the discussion (l. 681):

> Beside being closer to the normal operation of the whisker system, a further argument for why the readout should differentiate the local network activity is provided by our results shown in fig. 12 and discussed on p. 23: there, we introduced a proxy for slow modulations of the input to the network, i.e. a static input noise. Our results showed that, in this simplified situation, the readout performance of the integrator readout is more severely degraded than that of the differentiator. Although it remains to be seen whether this finding holds also for a true dynamical input noise, differentiating would be a strategy to the detector uses to deal with strong and slow variations in the network's firing state.

R #2: *--- Page 37 ---*
Can you discuss why the threshold crossing in from below for the IR and from above in the DN networks and maybe show an example in fig 3 of what that looks like?

The essential reason is that, in this way, the effect size is positive, as in the experiments. In the revised manuscript, we write (l.174):

> There is nothing profound in the choice to endow the IR with a lower detection boundary and the DR with an upper detection boundary. The essential reason is that, in this way, we obtain a positive *effect size* (defined below), as it was observed in the experiments. In other words, this combination works for the readout schemes we consider here.

R #2: *--- Page 43 ---*
Is the mean stimulus in fig9 symmetric about t=200?

The mean stimulus is the average over all possible permutations of the sequence of intervals. For each permutation, we can reverse the sequence and obtain another legitimate permutation, which is equally likely. Hence, the average stimulus must possess time-reversal symmetry, *i. e.* it is symmetric about its middle point (t=200). This fact is explained in the revised manuscript when fig. 9B is discussed (l. 483)

> The average signal is plotted in fig. 9B. By recalling that each irregular signal corresponds to one permutation of the step sequence, and recognizing that the reversed sequence is another valid *equally probable* permutation, one can conclude that the average signal must possesses time-reversal symmetry.

Reviewer #3:

Summary
Bernadi and colleagues – create a quasi-realistic model of barrel cortex, to understand how stimulation of individual cells, in turn impact network interactions and how the networks output can be read out by distinct read out classes (integrator versus differentiator).

We would like to thank the reviewer for the time and efforts devoted to our manuscript and for the appreciation of our results. We are grateful for the useful suggestions, which helped us improve the readout model and better illustrate the interpretation and significance of our results.

R #3: *Major Concerns*
Is the model focusing on what is happening in layer 4? It appears this way given the choice and proportion of neuronal phenotypes – be specific.

As already mentioned in the reply to Reviewer #2, we deliberately chose not to specify a single layer because in the experiments cells at different depths were stimulated. Furthermore, we can only speculate on where the readout population(s) may be located. With the exception of layer 1, the three neuron types we included in the model are present in high numbers all layers (see *e.g.* Meyer et al. PNAS 2011), although the relative proportion can indeed fluctuate. In this regard, we write (l.622):

> The spiking network model (BCN) we constructed in this study incorporates several features of the barrel cortex, and its parameters were consistent with the experimental literature. The BCN was chosen to represent only the immediate surroundings of the stimulated cell and its size is about one sixth of one standard cortical column [7]. Hence, our network neither has a layered structure (being too small), nor it represents one specific layer within one "cortical column". Indeed, there is no clear layer specificity from the experimental side, and, presumably, cells from different layers were stimulated. Also the readout part of our model represents a generic downstream area and may or may not be located within a specific layer, or within the barrel cortex itself. One hypothesis could be that the stimulated cell

resides in deeper layers and that the readout network is a located within the supragranular layers. At the present state, however, there is no specific element in support to this conjecture before others, including the possibility that the readout computation is not localized in a single population, but rather distributed across several downstream areas.

It is still unclear whether specific anatomical properties of the barrel cortex make it particularly sensitive to the single-neuron stimulation. In this regard, although we did use the available experimental literature on the rat barrel cortex as reference to choose the range in which all cellular parameters are heterogeneously distributed (alongside with connection probabilities and the types of synaptic plasticity), some of these characteristics are similar in other cortical areas, so that the general traits of the BCN could describe also other cortical systems. If this is the case, other cortical areas might be sensitive to the single-cell stimulation.

R #3: *Why assume the readout is only getting inputs from excitatory cells, if the model is representing layer 4 – then anatomical data suggests that both excitatory and inhibitory neurons within the layer 4-barrel synapse onto neurons in the supragranular and infragranular layers.*

Thank you for this suggestion. We have revised the model such that the readout now receives input also from inhibitory cells. As already outlined above in our response to Reviewer #1, the inclusion of input from inhibitory neurons improves the detection performance for reasons that are discussed in detail in the revised manuscript. Furthermore, we would like to emphasize that the revised ms contains a systematic investigation of how the readout performance depends on the number and type of neurons fed to readout, the results of which are summarized in the new fig. 10 and discussed in the main text as follows (l. 527):

After having shown that the DNR is most consistent with the experimental results, we will now explore how the readout performance depends on the size of the three readout populations, i.e. the effect of each neuron class on the detectability of the single-neuron stimulation. The simulation results are summarized in fig. 10. For brevity, we will focus on the IR (first row in fig. 10) and DNR (second row in fig. 10), and use only the 400 ms stimulus (represented by blue circles) and irregular stimulation (red squares).

First, we can systematically vary the size of the RS readout population. Intuitively, the effect size should increase, the more RS neurons are fed into the readout. Simulation results demonstrate that this is indeed the case both for the IR readout (fig. 10A) and the DNR readout (fig. 10B): the effect size grows monotonically with the size of the RS readout population.

Next, we can study the effect of a systematic variation of the FS readout population's size. In fig. 10C (D), it can be seen how the effect size measured by the IR (DNR) decreases when the FS readout population is enlarged. The strong self-inhibition of FS cells tends to stabilize their firing rate. Hence, despite the strong SOM response within the BCN, the average firing rate of FS cells within the BCN is, on average, only slightly reduced during the single-cell stimulation. However, due to the strong output weights of FS cells, they have a considerable effect on the readout activity. Because they disinhibit the readout during the stimulation, the effect of FS cells on the readout is antagonist to that of RS cells.

The readout performance is improved by feeding more SOM neurons into the IR (fig. 10E). The readout performance of the DNR is generally improved by enlarging the number of SOM readout cells (fig. 10F). However, the effect size

> tends to saturate (for irregular stimulation) or even slightly decrease when the size the readout population approaches the entire SOM population (200 cells). One possible reason may be stronger cross-correlations between SOM cells.
>
> As a final comment to fig. 10, we note that the DNR detects the irregular signal with a higher accuracy over the entire range of parameters, while the IR readout is almost always better at detecting the regular signal. In this respect, these findings are robust, and the DNR is a stronger candidate than the IR as a possible readout mechanism which is in line with the experimental results.

R #3: *The goal of the study is to describe the influence of a single neuron in most cases with a relatively short punctate stimulus, while a good starting point, it is not clear how useful this will be given the nature of inputs in natural settings which co-activate hundreds if not thousands of neurons within very short time windows.*

Theoretical arguments and experimental results suggest that the encoding in the cortex is sparse (see *e.g.* Wolfe et al. *Curr. Opin. Neurobiol.* 2010, 20), so that the number of neurons co-activated by natural stimuli may be not always excessively large. Still, the juxtacellular nanostimulation is a kind of stimulus that is indeed artificial. Nevertheless, the experimental finding that the stimulation of *one single neuron* in the barrel cortex can influence the behavior of an animal that is awake is a striking finding that contradicts most textbook assumptions on the neural coding, but that so far has received little attention in the theoretical and modeling literature. As we noted at the beginning of this reply, our previous studies are inconsistent with how the behavioral response depends on the properties of the injected stimulus, a void which we aim at filling with the present study.
In this regard, beside the passage in the introduction quoted on p.2 of this reply, we now write in the revised Discussion (l. 605):

> The development of juxtacellular stimulation has brought remarkable experimental opportunities, ranging from reliably evoking prescribed spike trains [52–54] to probing the role of a single neuron in the perception of sensory inputs and motor responses [2–6]. Although these experiments question the well-established belief that large numbers of neurons are needed to encode a stimulus or elicit a behavioral response, they have received little attention in the theoretical and modeling literature. Recently, first attempts were made to model the behavioral responses to the stimulation of a single cortical neuron in rodents [25, 26, 55]. However, how the behavioral response probability is influenced by the properties of the injected current [6, 48] is still an open theoretical challenge.

R #3: *Minor Issues*
Line 64 first use of BCN define the abbreviation at this point it is not defined in the text until line 70

This has been fixed, we apologize for the mistake.

***R #3:** Lines 67-68 2600 neurons might exist within a barrel or a barrel column but many more are within the barrel cortex itself.*

We apologize for this confusing formulation. We meant that, based on the current estimates for the average neuron *density* of the barrel cortex, 2600 neurons is a reasonable choice for the number of neurons contained in a sphere with a 200 μm radius. To avoid this misunderstanding, we rephrased this sentence and the next as follows (l. 70):

> The recurrent network model represents the surroundings of the stimulated cell in a radius of about 200 μm, in which connection probabilities can be considered as constant [27, 28]. Taking into account the estimated neuron density in the barrel cortex [7], a sphere with a radius of 200 μm contains about N = 2600 neurons, which we therefore take as the network size. Although this network size corresponds to a fraction of one barrel, and an even smaller fraction of the entire barrel cortex, this part of the model will be referred to as "barrel cortex network" (BCN);

Further changes:

After the first submission we became aware of a recent publication related to our topic. We cite and discuss this paper at the beginning of the revised discussion (l. 612):

> ...how the behavioral response probability is influenced by the properties of the injected current [6, 48] is still an open theoretical challenge. A recent computational study has demonstrated how irregular stimuli can be more effective in triggering a network burst than a regular stimulus [55]. However, the proposed mechanism does not provide an explanation for the weak sensitivity to the stimulus duration or intensity. In the present study, we constructed a model on different premises, in which the network keeps firing asynchronously and does not burst. Our model can successfully reproduce that the probability of the behavioral response is not substantially influenced by the duration or by the intensity of a constant stimulus, but it is strongly dependent on whether irregular or regular stimuli are used.

Further minor changes were needed to integrate the new results. We also added a few stylistic modifications and removed typos. All changes are marked in red in the revised ms.
