# Supplementary material for: A network model of the barrel cortex combined with a differentiator detector reproduces features of the behavioral response to single-neuron stimulation
Source: PLoS Comput Biol. 2021 Feb 8;17(2):e1007831. doi: 10.1371/journal.pcbi.1007831 (PMC7895413; doi:10.1371/journal.pcbi.1007831)
Supplement: Attachment — Submitted filename: PCOMPBIOL-D-20-00502-reply-to-reviewers-2.pdf [file pcbi.1007831.s002.pdf]

Submission to PLoS Computational Biology:

"A network model of the barrel cortex combined with a differentiator detector reproduces features of the behavioral response to single-neuron stimulation"

(PCOMPBIOL-D-20-00502)

Davide Bernardi, Guy Doron, Michael Brecht, Benjamin Lindner

Reply with list of changes

Dear Editors,

We would like to thank the Editors and Reviewers for their time and for the appreciation of our efforts. Below, we list the changes we made to our manuscript according to the reviewers' suggestions. We provide also a revised manuscript in which all changes to the text are highlighted in red.

> ***Reviewer #1****:*
>
> *I thank the authors for their point-by-point reply to my comments. You have done a good job addressing my concerns in your revised work. I have mostly minor comments at this point, as detailed below.*
>
> *Minor comments:*
> *Line 133: The third readout scheme (fig. 2C) *is* based on the summed activity*

This was corrected, thank you.

> *Line 149: just to be clear, you mean that all dynamic weights J^e_read,i(t), J^i_read,i(t), and J^s_read,i(t) are depressing? please clarify.*

These weights are all depressing. To clarify this point, the paragraph has been edited as follows (l.147-153):

> Consistent with the idea that this readout is performed by other brain areas, and that synapses projecting to other brain areas are likely to undergo short-term depression, the dynamic weights $J^e_{read,i}(t)$, $J^i_{read,i}(t)$, and $J^s_{read,i}(t)$ are depressing and obey the same equation as their counterparts within the BCN, i.e. eqs. (34) and (35); STD parameters are randomly distributed as those within the BCN, and depend on the cell type, so that $J^e_{read,i}(t)$ and $J^i_{read,i}(t)$ are more strongly depressing than $J^s_{read,i}(t)$ (see Methods).

> *Fig.3 legend: it is unclear what the vertical dashed lines at 0 ms and 600 ms indicate. I*

assume this is your "detection time window" [0,T_w] as mentioned in the main text, but this should be clarified in the legend.

Indeed, the vertical dotted lines at 0 ms and 600 ms indicate the detection time window. This is now explicitly indicated in the figure caption.

Fig.4: on the figure itself, should delta_r be changed to delta_r_e to reflect the fact that the DRN perturbs RS neurons from the BCN?

Fig. 4 is correct but the first sentence of the caption was not properly edited during the last revision. The corrected caption reads now:

> **Fig 4. Tuning of the differentiator readout network to implement the operation of the differentiator readout scheme.** A perturbation in the firing rate of the BCN (Δr) causes a perturbation in the mean input to the RS readout neurons...

Further, to facilitate reading, I would suggest that the "strength of the connection from I to S^B" mentioned in the figure legend be followed by "(J^R_ei)".

This was inserted as suggested.

Fig.5 legend: " as it can be seen by excluding" remove "it" (same comment for line 388).

Both sentences were changed as suggested.

Line 442: The sentence beginning with "As the most prominent features..." seems to be incomplete. I believe that what you mean is that there is a null net effect on effect size?

The last part of the sentence was indeed missing, thank you for pointing this out. The sentence reads now (l. 445-447 in the revised manuscript):

> As the most prominent features of $\hat{\mu}_{dr}(t)$ have opposing effects and become stronger simultaneously upon growing stimulus intensity, the net effect on the effect size is barely noticeable (fig. 8G).

Fig.9, Panel A: the y-axis label should be "Rel. current intensity".

Fig.9 has been edited and the typo has been removed.

Fig.12, legend: "filtered with with a 1 s sliding window" remove "with"
Line 582: "justacelluar" should be "juxtacelluar"

Line 688: " differentiating would be a strategy to the detector uses": replace "to" with "that".

These three mistakes have been corrected, thank you.

Discussion: I was surprised not to see a reference to the work of Mainen and Sejnowski 1995 Science, as they were amongst the first to discuss cortical responses to constant vs. irregular stimulation and its relation to spike reliability. Perhaps I am just getting old, but it would be nice to see at least a quick mention of this paper (perhaps around line 640 regarding general mechanisms across cortical areas?)

We make a reference to this classic paper in the revised Discussion (l. 647):

> The increased response probability for an irregular stimulation and the weak sensitivity to a step-current bring to mind the much-debated observation that irregular stimuli increase the response consistency of a single cortical cell *in vitro* [56], which was recently observed also *in vivo* [52]. These studies, however, considered the reliability in the spike sequence generated by a single cortical cell in response to repeated presentation of a frozen stimulus, and not the response to randomized stimuli at the behavioral level. Hence, it was still a completely open question what kind of mechanisms could be involved in the response of the local network and in the subsequent processing stages to regular and irregular stimuli. Among the biological processes included in the model, short-term depression and spike frequency adaptation could be expected to oppose slow changes in the input. However, our results indicate that these mechanisms may not suffice to explain the data if an integrator readout (IR) is employed. If a differentiator readout (DR or DNR) is used instead...

***Reviewer #2**: I was not able to read this easily as the figures are not in the main body of the text and are not even with the captions. It seems that you have answered my objections for the most part*

***Reviewer #3:** the authors have effectively answered my concerns*

Further changes:

We noticed that the shades of blue of SOM cells' output connections in the drawings of Fig.1 and Fig.2 were not entirely consistent. This has been fixed to prevent misunderstandings. For the same reason, the last sentence of the caption of Fig. 2 has been changed as follows:

> All connections depicted in blue and light blue are dynamic and show short-term depression (STD).

One further typo on l.662 has been corrected.
